# Infant gut microbiota and the development of wheeze in early childhood

**DOI:** 10.1186/1710-1492-10-S1-A35

**Published:** 2014-03-03

**Authors:** Scarlet M Salas, Meghan B Azad, Tedd Konya, David S Guttman, Allan B Becker, Malcolm R Sears, James A Scott, Anita L Kozyrskyj

**Affiliations:** 1 Manitoba Institute of Child Health, Winnipeg, Manitoba, R3E 3P4, Canada; 2 Pediatrics and Child Health, University of Manitoba, Winnipeg, Manitoba, R3E 3P4, Canada; 3 Pediatrics, University of Alberta, Edmonton, Alberta, T6T 1C9 Canada; 4 Dalla Lana School of Public Health, University of Toronto, Ontario, M5T 1R4, Canada; 5 Cell & Systems Biology, University of Toronto, Toronto, Ontario, M5S 3B2, Canada; 6 Medicine, McMaster University, Hamilton, Ontario L8S 4K1, Canada; 7 Canadian Healthy Infant Longitudinal Development Study, Canada

## Background

Commensal gut microbes play an important role in human development and lifelong health. There is increasing evidence that disruption of the infant gut microbiota may be linked to the development of childhood allergy and asthma-related outcomes, including wheezing. Our objective was to investigate the association of infant gut microbiota diversity and composition with development of wheeze in early childhood.

## Methods

The study population included 160 infants enrolled at the Winnipeg site of the Canadian Healthy Infant Longitudinal Development Study (CHILD) population-based birth cohort. Standardized questionnaires were completed by mothers at 3, 6 and 12 months after birth, and reported on breastfeeding and occurrence of infant wheezing. Wheezing (defined as a whistling sound in the chest lasting more than 15 minutes and occurring with or without a cold) was classified according to the number of episodes in the first year of life: 0, 1, 2 or ≥3. Mode of delivery and use of antibiotics were documented from hospital and medical records. Fecal samples were collected at one year of age and microbiota composition (relative abundance of select taxa) was characterized by high-throughput Illumina sequencing of the 16S rRNA gene. Biodiversity was evaluated using the Chao1 richness estimator and the Shannon & Simpson diversity indices.

## Results

In the first year of life, any wheezing as defined above was reported for 36/160 infants (22.5%). Sixteen infants (10.0%) wheezed on more than one occasion, and 12 infants (7.5%) experienced 3 or more wheezing episodes. Selected taxa were chosen for composition analysis, based on their established associations with other allergic disease outcomes. The genus Clostridium was under-represented among infants with two or more wheezing episodes (p=0.04). Preliminary comprehensive analyses revealed that operational taxonomic units (OTUs) belonging to the Families Ruminococcaceae, Rikenellaceae and Lachnospiraceae were over-represented among wheezing infants (all p<0.01). Gut microbiota diversity and richness were not significantly associated with wheeze; these findings were unchanged following adjustment for gender, mode of delivery, breastfeeding, and antibiotic exposure.

## Conclusions

In this preliminary analysis, we identified several crude differences in microbiota composition among wheezing infants, although a significant association with overall biodiversity was not observed. The long-term clinical relevance of these changes will be the focus of ongoing studies within the CHILD cohort. Detailed and extended assessment of wheeze (including its severity and association with infection and allergic disease), gut microbiota, and relevant environmental exposures will be conducted at different ages throughout childhood.

